# Macrophage Migration Inhibitory Factor and Post-Discharge Inflammatory Profiles in Severe COVID-19: A Prospective Observational Study from Romania

**DOI:** 10.3390/ijms26199697

**Published:** 2025-10-05

**Authors:** Nimród László, Corina Mărginean, Botond Barna Mátyás, Cristina Alexandra Man, Előd Ernő Nagy, Gabriela Jimborean

**Affiliations:** 1 Department of Pulmonology, “George Emil Palade” University of Medicine, Pharmacy, Science, and Technology of Târgu Mureș, 540142 Târgu Mureș, Romania; nimrod.laszlo@umfst.ro (N.L.); gabriela.jimborean@umfst.ro (G.J.); 2 Doctoral School of Medicine and Pharmacy, “George Emil Palade” University of Medicine, Pharmacy, Science, and Technology of Târgu Mureș, 540139 Târgu Mureș, Romania; matyas_botond@yahoo.com (B.B.M.); cristina.man@umfst.ro (C.A.M.); 3 Department of Cardiology, “George Emil Palade” University of Medicine, Pharmacy, Science, and Technology of Târgu Mureș, 540142 Târgu Mureș, Romania; 4 Department of Biochemistry, “George Emil Palade” University of Medicine, Pharmacy, Science, and Technology of Târgu Mureș, 540142 Târgu Mureș, Romania; elod.nagy@umfst.ro

**Keywords:** COVID-19, cytokines, MIF, IFN-γ, TNF-α, immune recovery, Romania, long COVID

## Abstract

Dysregulated cytokine responses are a hallmark of severe COVID-19; however, the persistence of these responses following hospital discharge remains inadequately understood. This study aimed to characterize the inflammatory profile of hospitalized COVID-19 patients in Mureș County, Romania, at the point of admission and one month post-discharge. We conducted a prospective observational study involving 68 patients with RT-PCR-confirmed SARS-CoV-2 infection, classified according to disease severity. Blood samples were collected at baseline and after one month. Macrophage migration inhibitory factor (MIF) levels were quantified using ELISA, while other cytokines, including MCP-1, IP-10, IFN-γ, IL-4, IL-10, IL-13, IL-17, and TNF-α, were measured via Luminex multiplex assays. Patients with severe disease exhibited significantly elevated levels of MIF, IFN-γ, IL-17, and TNF-α at admission (*p* < 0.0001). Although cytokine concentrations generally declined over time, patients with severe disease continued to display persistently elevated MIF (mean 31,035 pg/mL), IFN-γ, and TNF-α, indicative of ongoing inflammatory processes. Clinical parameters such as respiratory rate and oxygen saturation correlated with disease severity. These findings suggest that severe COVID-19 induces a prolonged inflammatory response, with MIF and IFN-γ remaining elevated beyond the acute phase. Cytokine profiling holds potential for improving prognostic assessments and identifying patients at risk of long-term immune dysregulation, with MIF emerging as a potential candidate marker for immune recovery and a possible target for therapy.

## 1. Introduction

Since its emergence in late 2019, coronavirus disease 2019 (COVID-19), caused by severe acute respiratory syndrome coronavirus 2 (SARS-CoV-2), has greatly challenged healthcare systems and scientific research worldwide. While most infected individuals experience mild to moderate symptoms, a notable proportion develop severe illness marked by respiratory failure, systemic inflammation, and multi-organ dysfunction, often necessitating hospitalization and intensive care. A key factor contributing to this severe disease progression is an overactive immune response, commonly known as the “cytokine storm,” which involves elevated levels of both pro-inflammatory and anti-inflammatory mediators in the bloodstream. The complex interplay between systemic and local inflammatory responses has also been highlighted in cardiovascular diseases, where such immune activation was shown to influence clinical outcomes after myocardial infarction [1].

Several studies have identified specific cytokines and chemokines, such as interleukin-6 (IL-6), tumor necrosis factor-alpha (TNF-α), interferon-gamma (IFN-γ), monocyte chemoattractant protein-1 (MCP-1), and interleukin-10 (IL-10), as key biomarkers associated with COVID-19 severity and prognosis [2,3,4,5]. However, data on longitudinal immune changes during the recovery phase, especially in moderate and severe cases, remain limited. Additionally, real-world clinical data from Eastern European countries, particularly Romania, are markedly underrepresented in the global COVID-19 literature despite high hospitalization and mortality rates during pandemic waves [6,7]. We recently showed that COVID-19 is associated with increased coronary inflammation, detectable by CT-derived epicardial fat attenuation, in a Romanian cohort [8].

During the study period (January–June 2022), Romania reported some of the lowest COVID-19 vaccination rates in the European Union, with national coverage estimated at 42–45% and about 30% in our region [9]. This limited uptake contributed to sustained hospitalization rates as the transition occurred from the Delta wave in late 2021 to the Omicron BA.1/BA.2 wave in early 2022. National surveillance confirmed community transmission of Omicron from December 2021, and sequencing data indicated that by the first half of 2022, the majority of circulating strains in Romania were Omicron subvariants (BA.1 and BA.2), gradually replacing Delta [10].

Clinical data from Romanian cohorts further showed that unvaccinated individuals were significantly more likely to require hospitalization compared to those vaccinated, underscoring the increased burden on healthcare systems during this wave [10].

The cytokines chosen for analysis in this study were selected for their established involvement in COVID-19 immunopathology. IL-6, TNF-α, and IFN-γ are key pro-inflammatory mediators consistently involved in the cytokine storm and linked to negative clinical outcomes [11,12,13]. MCP-1 and IP-10 are important chemokines that regulate leukocyte recruitment, and their increased levels have been associated with pulmonary inflammation and tissue damage. IL-4, IL-10, and IL-13 represent anti-inflammatory and regulatory pathways, and maintaining a balance between these and pro-inflammatory cytokines is crucial for disease resolution. IL-17 indicates Th17-driven responses, which may impact post-infectious recovery and the persistence of immune activation [4,14].

Particular emphasis was placed on macrophage migration inhibitory factor (MIF), a multifunctional and pleiotropic cytokine that plays a central role in regulating innate and adaptive immunity. Initially described as a lymphokine that inhibited macrophage migration, MIF is now recognized as an upstream mediator of inflammation with broad biological effects. It is constitutively expressed in preformed intracellular pools and can be rapidly released by a variety of immune, endocrine, and epithelial cells, including T lymphocytes, macrophages, dendritic cells, neutrophils, pituitary cells, and vascular endothelial cells [15,16,17]. Once secreted, MIF coordinates leukocyte trafficking and amplifies inflammatory cascades through the induction of other mediators such as TNF-α, IL-1β, and IFN-γ. Additionally, it promotes endothelial dysfunction, contributing to the hyperinflammatory environment characteristic of severe systemic illness. Elevated circulating levels of MIF have been consistently reported in sepsis and acute respiratory distress syndrome, where they correlate with clinical severity and adverse outcomes [18,19,20]. In COVID-19, MIF has received growing attention as a marker of disease severity, and preliminary studies suggest it may be explored as a therapeutic target. However, its longitudinal profile after hospitalization remains insufficiently characterized, and prospective data in this setting are limited.

To address this gap, we designed a prospective observational study enrolling hospitalized COVID-19 patients from two tertiary centers in Mureș County, Romania. Our aim was to characterize longitudinal changes in circulating cytokines by comparing immune profiles at admission and at one month after discharge. We specifically sought to delineate baseline inflammatory signatures across disease severity strata and to evaluate whether post-hospitalization changes reflected recovery or persistent immune activation. We hypothesized that patients with severe COVID-19 would exhibit persistently elevated cytokine levels, with particular emphasis on MIF, compared with those with mild or moderate disease.

## 2. Results

### 2.1. Patient Characteristics

A total of 68 hospitalized COVID-19 patients were included in the final analysis, all admitted between January and June 2022, having completed both baseline and 1-month follow-up assessments. All patients were unvaccinated at the time of admission. Patients were divided into three severity groups at admission according to WHO clinical management criteria [21,22]: mild (*n* = 16; no pneumonia or hypoxia, SpO_2_ ≥ 90% on room air), moderate (*n* = 28; clinical or radiographic pneumonia with SpO_2_ ≥ 90% on room air, no signs of severe disease), and severe (*n* = 24; SpO_2_ < 90% on room air, respiratory rate > 30 breaths/min, severe respiratory distress, or requirement for supplemental oxygen/ICU admission; severe and critical cases were merged into a single ‘severe’ group for analysis). No significant differences were observed in age (mean 69.9 ± 12.4 years), gender distribution (58.8% male), or BMI (mean 29.87 ± 3.9 kg/m^2^) among the three groups (Table 1).

Comorbidities including hypertension, cardiovascular disease, obesity, diabetes mellitus, COPD, and asthma were similarly distributed across severity groups, with no statistically significant differences. Fever was the most common presenting symptom (85.3%), followed by cough (35.3%) and expectoration (29.4%).

Physiological markers such as respiratory rate and oxygen saturation showed significant trends across severity groups. Respiratory rate increased significantly from mild to severe disease (20.1 ± 2.1 vs. 25.1 ± 3.1 breaths/min, *p* < 0.0001), while oxygen saturation declined significantly with increasing severity (96.3 ± 2.4% in mild vs. 82.2 ± 6.4% in severe cases, *p* < 0.0001) (Table 1).

### 2.2. Baseline Cytokine Profiles

At hospital admission, MIF levels were found to be significantly elevated in patients with increasing disease severity. Specifically, patients classified as mild exhibited levels of 18,896 ± 5202 pg/mL, those with moderate disease had levels of 31,590 ± 5179 pg/mL, and individuals with severe disease had levels reaching 51,930 ± 5511 pg/mL. The differences across these groups were highly significant (*p* < 0.0001), indicating a positive correlation between MIF levels and disease severity.

**Figure 1:**
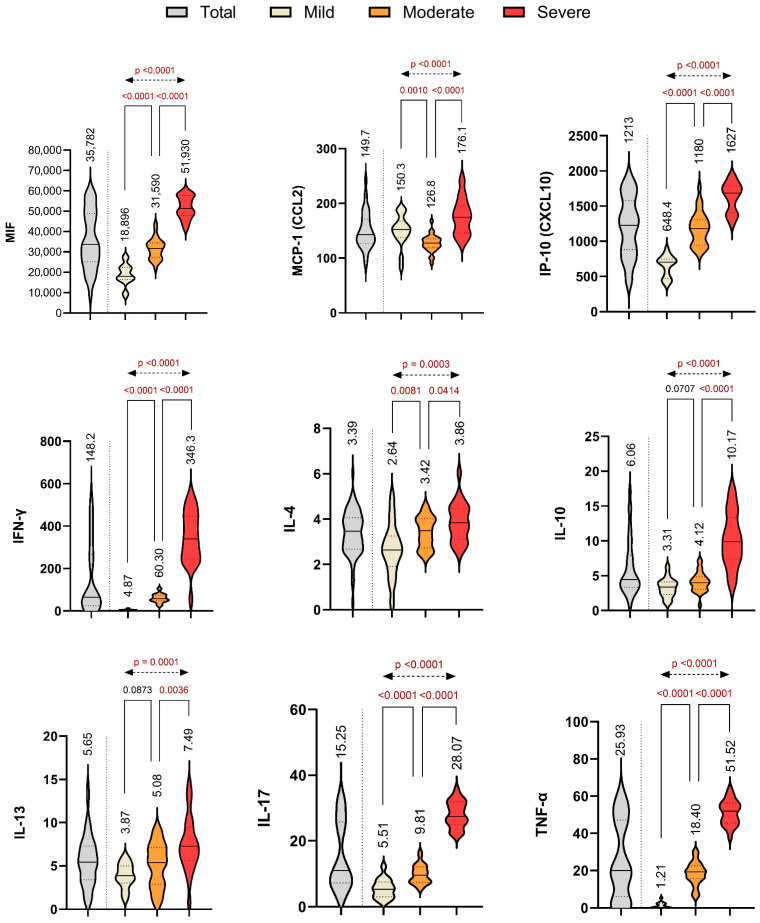
Baseline serum cytokine levels and inflammatory markers among patients stratified by disease severity. Violin plots illustrate the distribution of MIF, MCP-1, IP-10, IFN-γ, IL-4, IL-10, IL-13, IL-17, and TNF-α across the three severity groups at baseline. Median values are indicated, and intergroup comparisons are annotated with significance levels (*p* values). Statistical analysis was performed using one-way ANOVA for continuous variables with Tukey’s HSD post hoc test, and Chi-square test for categorical variables, with Fisher’s exact test and Bonferroni correction applied to pairwise comparisons (α = 0.0167).

Similarly, levels of MCP-1, IP-10, IFN-γ, IL-4, IL-10, IL-13, IL-17, and TNF-α were significantly elevated in severe cases compared to mild and moderate groups (Table 2, Figure 1). IFN-γ, TNF-α, and IL-17 showed especially pronounced increases in the severe group (all *p* < 0.0001), highlighting their link to COVID-19-related hyperinflammation.

### 2.3. Cytokine Levels at 1-Month Follow-Up

**Figure 2:**
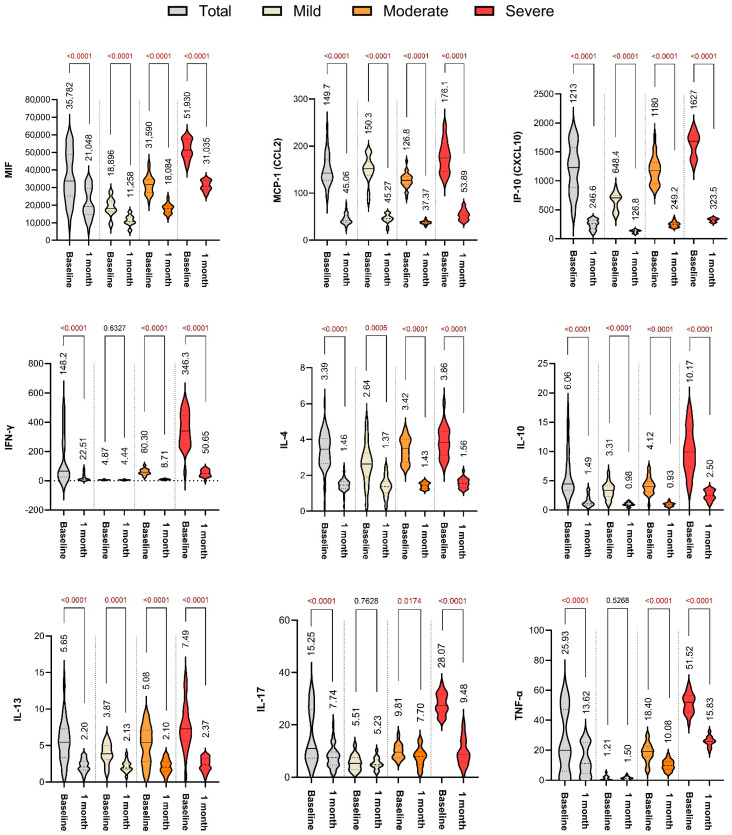
Longitudinal changes in cytokine and inflammatory marker concentrations at 1-month follow-up compared to baseline. Paired violin plots illustrate reductions in MIF, MCP-1, IP-10, IFN-γ, IL-4, IL-10, IL-13, IL-17, and TNF-α over time in the entire cohort and within each severity group. Statistical comparisons show significant post-treatment decreases across most markers.

One month after discharge, a significant decrease in cytokine levels was observed across all participant groups. Despite these declines, levels remained higher in the previously severe group compared to the mild and moderate groups, indicating persistent immune activation. Specifically, MIF levels decreased from 51,930 ± 5511 to 31,035 ± 2968 pg/mL in the severe group (*p* < 0.0001). Similarly, IFN-γ levels declined sharply from 346.3 ± 121.1 to 50.65 ± 23.16 pg/mL (*p* < 0.0001), and TNF-α levels dropped from 51.52 ± 6.62 to 25.83 ± 3.42 pg/mL (*p* < 0.0001). Despite these reductions, levels of MCP-1, IL-17, and IL-10 continued to be significantly elevated in the severe group compared to the other groups, suggesting that immune recovery remained incomplete. (Figure 2, Table 3).

At the 1-month follow-up visit, 18% of patients reported persistent symptoms. The most common were fatigue, cognitive difficulties (“brain fog”), shortness of breath, chest pain, arthralgia/myalgia, insomnia, and post-exertional malaise. However, the presence of these symptoms did not show a significant correlation with the severity of the acute illness.

### 2.4. Summary of Immune Resolution Trends

Paired analyses confirmed statistically significant declines in all cytokines over time in the total cohort (all *p* < 0.0001 for MIF, IFN-γ, TNF-α, IP-10, IL-17). However, the reduction was less pronounced in the severe group compared to mild or moderate cases, supporting the hypothesis of prolonged immune dysregulation after severe COVID-19 infection (Figure 2).

## 3. Discussion

Our study presents a detailed immunological profiling of hospitalized COVID-19 patients in Romania, highlighting significant differences in cytokine responses based on disease severity and their evolution over time. The findings confirm that elevated inflammatory mediators, especially MIF, IFN-γ, TNF-α, and IL-17, are associated with increased clinical severity, while also showing that systemic inflammation often persists even one month after hospital discharge in patients with severe disease.

These results are consistent with prior research indicating that a dysregulated cytokine environment is central to COVID-19 pathogenesis. Studies have repeatedly shown that severe COVID-19 is characterized by a hyperinflammatory immune response often referred to as a “cytokine storm,” involving a wide array of pro-inflammatory and anti-inflammatory mediators, including IL-6, TNF-α, and IFN-γ [11,12,13]. While much of the global literature has focused on cytokine increases during the acute phase, few studies have looked at immune recovery after discharge. Our findings contribute to this understanding by showing that cytokine dysregulation, particularly in MIF and IFN-γ, may persist even after 4 weeks of clinical improvement.

### 3.1. MIF: A Key Mediator of COVID-19 Severity and Persistence

Among the most notable findings in our study is the consistent and significant increase in MIF in severe cases both at admission and during follow-up. MIF has a complex role in regulating innate and adaptive immunity and is known to drive endothelial activation, oxidative stress, and leukocyte recruitment [15]. Previous studies have shown that higher MIF levels can predict mortality in sepsis and acute respiratory distress syndrome (ARDS) [18,19,20], and emerging COVID-19 data confirm its role in disease development [16,17]. MIF modulates CD8+ T cell survival and cytokine production in severe COVID-19, suggesting it is a key regulator of immune imbalance. Our findings support earlier evidence and further suggest that MIF stays elevated after hospital discharge, raising the possibility of its involvement in long COVID development [19]. This idea needs to be tested in larger, longer-term studies.

### 3.2. Persistent Cytokine Activation After Severe COVID-19

A key observation from this study is that patients with severe COVID-19 show evidence of incomplete immune recovery even one month after discharge. Although most cytokine levels decrease significantly from their peak values, MIF, IFN-γ, IL-10, IL-17, and TNF-α remain elevated in patients who previously had severe patients compared to those with mild or moderate disease. These results align with earlier reports of ongoing immune activation in COVID-19 survivors [23,24]. For example, Laing et al. [3] noted persistent elevations in IFN-γ and TNF-α in recovered patients with severe illness, which may contribute to chronic fatigue, pulmonary fibrosis, and other post-viral sequelae. Similarly, Del Valle et al. [13] and Lucas et al. [14] found that the trajectory of cytokine decline correlates with clinical recovery, and incomplete resolution is often seen in cases that develop post-acute sequelae (PASC). Our findings support this evidence, suggesting that IL-17 and MIF could be potential markers of incomplete immune recovery and risk of long COVID. Further research with larger and longer-term studies is needed to confirm this [25,26].

In our cohort, nearly one in five patients reported persistent symptoms at 1-month follow-up, but these were not significantly associated with acute disease severity. This aligns with previous reports showing that although the risk of long COVID increases with more severe illness, persistent symptoms can also occur after mild infections [27]. The lack of correlation in our study might be due to the relatively small sample size and highlights the multifactorial nature of post-acute sequelae, in which immune dysregulation is just one of several contributing mechanisms.

### 3.3. Comparison with Regional and Global Data

To date, Eastern European populations, including Romania, have been underrepresented in global COVID-19 biomarker studies [28]. The cytokine signatures observed in our cohort are broadly consistent with those reported in cohorts from China [29], Western Europe [3,14], and the United States [2,13]. The persistently high post-discharge MIF levels observed in our cohort may reflect several interacting factors. These include a higher prevalence of undiagnosed comorbidities, delayed hospital presentation, and genetic variability in MIF-related pathways (e.g., CD74 polymorphisms) [16]. In addition, patients were recruited from three counties in central Romania during a period of marked healthcare strain, when ICU capacity was frequently exceeded and access to advanced antiviral or immunomodulatory therapies was limited. Such regional healthcare constraints may have contributed to amplified inflammatory responses and slower post-discharge recovery. These hypotheses remain speculative and require validation in larger, multinational cohorts with extended follow-up.

### 3.4. Clinical Implications

Our findings have several potential clinical implications. First, MIF and other cytokines may have potential as prognostic indicators at admission and, if validated, may help inform early risk stratification and guide future development of targeted immunomodulatory approaches. Second, the persistence of elevated MIF, IFN-γ, and IL-17 at one-month follow-up suggests that patients recovering from severe disease could be considered for closer post-discharge monitoring, although the clinical advantages of anti-inflammatory interventions in this setting require further investigation. Lastly, the immunological profile described may highlight therapeutic pathways that merit further investigation in future clinical studies, potentially including those involving MIF or downstream cytokine signaling mechanisms.

### 3.5. Future Directions

These findings should be interpreted in light of several limitations. The relatively small, single-region cohort and exclusion of vaccinated patients limit generalizability, and survival bias may have underestimated inflammatory persistence in the most severe cases. In addition, the lack of viral load or variant sequencing and the use of a single 1-month follow-up point restrict the scope of our conclusions. Finally, the absence of a healthy control group limits interpretation of whether cytokine levels fully normalized during recovery. Future research should aim to validate these observations in larger, multicenter cohorts with extended follow-up to clarify the mechanisms underlying persistently high MIF levels and their clinical implications.

## 4. Materials and Methods

### 4.1. Study Design and Setting

This was a prospective, observational cohort study conducted between January to June 2022 at two tertiary hospitals in Mureș County, Romania: the Infectious Diseases Hospital and the Pulmonology Clinic. The study aimed to examine longitudinal changes in circulating cytokine profiles among hospitalized COVID-19 patients, with a particular focus on markers linked to disease severity and recovery.

### 4.2. Patient Population

Hospitalized adult patients (≥18 years) with confirmed SARS-CoV-2 infection by reverse transcription polymerase chain reaction (RT-PCR) were eligible for inclusion. Patients were enrolled within 48 h of hospital admission. Key exclusion criteria included the following: (1) death before 1-month follow-up, (2) pre-existing immunosuppressive therapy, (3) active malignancy, or (4) inability to provide informed consent. All participants provided written informed consent prior to inclusion. The study protocol was approved by the institutional ethics committees of both participating hospitals (Approval No. 38/2022) and adhered to the Declaration of Helsinki.

Because vaccination has been shown to alter systemic inflammatory responses and attenuate cytokine storm, vaccinated individuals were excluded to prevent introducing biological heterogeneity into the cytokine analyses. During the study period, overall COVID-19 vaccination coverage in Romania remained low (42–45% nationally and approximately 30% in the study region), and among patients hospitalized at our centers, fewer than 10% had received vaccination. Of the patients who were otherwise eligible for inclusion, a total of five vaccinated individuals were excluded for this reason, resulting in a final cohort composed entirely of unvaccinated patients.

Patients were divided into three groups based on the WHO clinical classification of COVID-19 severity at admission. The first group, labeled Mild, included patients with no pneumonia or hypoxia who received supportive care. The second group, Moderate, consisted of individuals showing radiographic evidence of pneumonia and/or maintaining an oxygen saturation (SpO_2_) of 90% or higher on room air. The third group, Severe, consisted of patients presenting with an SpO_2_ below 90% on room air, a respiratory rate exceeding 30 breaths per minute, or those requiring supplemental oxygen or intensive care. Peripheral blood samples were collected at two distinct time points: initially within 48 h of admission (baseline), and subsequently at approximately one month post-discharge (±3 days) during an outpatient visit. Baseline samples were obtained prior to initiation of in-hospital pharmacological treatment to prevent confounding by therapy. Serum was separated by centrifugation and stored at −80 °C until batch analysis. Only patients who provided samples at both time points were included in the final analysis.

### 4.3. Cytokine Quantification

Therapy was administered according to the hospital COVID-19 management protocol in effect during early 2022, tailored to the previously established severity groups. Patients with mild disease received only supportive care, including antipyretics, hydration, and clinical monitoring, without corticosteroids, anticoagulation, or antiviral therapy. Moderate cases were treated with weight-adjusted low-molecular-weight heparin for thromboprophylaxis, gastric protection with omeprazole, and systemic corticosteroids (typically dexamethasone 6 mg daily or equivalent), while Favipiravir (200 mg) was administered according to protocol criteria and drug availability. Severe cases received systemic corticosteroid therapy (dexamethasone 6 mg daily or equivalent), weight-adjusted low-molecular-weight heparin, and gastric protection with omeprazole. Favipiravir (200 mg) was administered as indicated, and antifungal prophylaxis with fluconazole was routinely given.

A panel of nine inflammatory mediators was quantified: MIF, MCP-1 (CCL2), IP-10 (CXCL10), IFN-γ, IL-4, IL-10, IL-13, IL-17, and TNF-α.

MIF Quantification: Serum macrophage migration inhibitory factor was measured using the Quantikine^®^ Human MIF ELISA (R&D Systems, Minneapolis, MN, USA) following the manufacturer’s instructions. The assay sensitivity was 16 pg/mL on average (range, 5–68 pg/mL), with a validated dynamic range of 160–30,000 pg/mL. Reported intra-assay coefficients of variation ranged from 4% to 6.5%, and inter-assay CVs from 6.5% to 8%, indicating high reproducibility. No significant cross-reactivity with related analytes was observed. All samples were analyzed in duplicate, and absorbance was read at 450 nm using a BioTek microplate reader (BioTek Instruments, Winooski, VT, USA).

Multiplex Cytokine Analysis: MCP-1, IP-10, IFN-γ, IL-4, IL-10, IL-13, IL-17A, and TNF-α were quantified simultaneously using a Human Magnetic Luminex^®^ Discovery Assay (Bio-Techne/R&D Systems) on a Luminex 200 system (Luminex Corp., Austin, TX, USA). The minimum detectable concentrations were: MCP-1, 0.53 pg/mL; IP-10, 4.1 pg/mL; IFN-γ, 1.12 pg/mL; IL-4, 0.39 pg/mL; IL-10, 2.2 pg/mL; IL-13, 1.14 pg/mL; IL-17A, 0.31 pg/mL; TNF-α, 0.18 pg/mL. Intra-assay CVs were consistently below 10%, and inter-assay CVs below 15% for all analytes, with no significant cross-reactivity reported. Calibration and quality control were performed using highly purified recombinant human cytokine standards. All standards and samples were analyzed in duplicate, and data were acquired and processed using xPONENT software v4.2.

### 4.4. Clinical and Demographic Data

Demographic, clinical, and laboratory data were extracted from electronic medical records. This included age, sex, body mass index (BMI), comorbidities, presenting symptoms, physiological parameters, and length of hospital stay. Oxygen saturation and respiratory rate were measured on room air at baseline. Data were anonymized prior to analysis.

Persistent symptoms at one month were assessed through a structured clinical interview during the outpatient follow-up visit, following institutional post-COVID procedures at both hospitals. Patients were systematically asked about respiratory, constitutional, and neurocognitive complaints, as well as any new or ongoing symptoms since discharge. This standardized approach ensured consistency across all participants. To evaluate differences in the prevalence of persistent symptoms between groups, we used the chi-square test. The analysis did not find a statistically significant association between patient group and the presence of persistent symptoms at one month (*p* = 0.41).

### 4.5. Statistical Analysis

Continuous variables were expressed as mean ± standard deviation (SD) or as median with interquartile range (IQR), as appropriate. Categorical variables were summarized as absolute counts and percentages. Normality of continuous variables was assessed using the Shapiro–Wilk test prior to applying parametric tests; non-parametric tests were applied when distributions were non-normal. Overall group comparisons for continuous variables were conducted using one-way analysis of variance (ANOVA), followed by Tukey’s honestly significant difference (HSD) Post Hoc test for pairwise comparisons. For non-normally distributed variables, the Kruskal–Wallis test was used with Dunn’s Post Hoc correction. Categorical variables were compared using the Chi-square (χ^2^) test, and Fisher’s exact test was applied when expected cell counts were low. Bonferroni correction was applied to multiple pairwise comparisons of categorical variables, with an adjusted significance threshold of α = 0.0167. Paired comparisons between baseline and one-month follow-up cytokine levels were assessed using paired *t*-tests for normally distributed variables or the Wilcoxon signed-rank test for non-parametric data.

Sample size justification: The study was exploratory in nature, and the sample size was determined on the number of eligible patients admitted during the study period who met the inclusion criteria and completed follow-up. Recruitment was further constrained by the limited hospital’s bed capacity during the peak of the pandemic, which restricted the number of patients that could be prospectively enrolled. No formal A Priori power calculation was performed. Given the modest cohort size, the analyses were primarily descriptive and hypothesis-generating.

Given the modest cohort size and uneven subgroup distribution, regression and multivariate analyses were not performed. Future larger studies will be required to identify independent predictors and to evaluate correlations between cytokine levels and clinical outcomes.

A two-tailed *p*-value < 0.05 was considered statistically significant. All statistical analyses were performed using GraphPad Prism version 10.3.1 (GraphPad Software, Inc., San Diego, CA, USA) and IBM SPSS Statistics version 26 (IBM Corp., Armonk, NY, USA).

## 5. Conclusions

This prospective cohort study of hospitalized COVID-19 patients from Romania provides evidences that cytokine dysregulation, particularly involving MIF, IFN-γ, TNF-α, and IL-17, is associated with disease severity and may persist into the convalescent phase in severe cases. Our findings suggest that MIF may hold potential as a candidate biomarker for acute risk stratification and post-discharge immune monitoring, although validation in larger, multicenter cohorts with longer-term follow-up is needed.

Importantly, the observed persistence of inflammatory markers one month after hospitalization emphasizes the importance of structured follow-up, while the possible role of targeted anti-inflammatory interventions requires further investigation. These data also support the view that post-acute sequelae, or “long COVID,” may be linked to prolonged immune activation.

### Study Limitations

Despite the strengths of our prospective design and dual time point cytokine profiling, several limitations should be acknowledged:

1. Single-country, single-region population: All patients were recruited from Mureș County, Romania, which might limit how well the results apply to populations with different genetic backgrounds, healthcare systems, or treatment protocols.
2. Small cohort size and survival bias: The total cohort of 68 patients enabled comparisons across severity groups, but subgroup sizes were modest, particularly for mild cases (*n* = 16). Furthermore, patients who died before the 1-month follow-up were excluded, introducing survival bias and potentially underestimating persistent inflammation in the most severe cases.
3. Lack of viral load and variant data: SARS-CoV-2 viral RNA was not quantified, nor were circulating variants sequenced. These details could have affected cytokine responses, especially considering the changing variant landscape during early 2022.
4. Potential residual confounding: Although patients receiving immunosuppressive therapy or with active malignancy were excluded, common comorbidities such as obesity, diabetes, and COPD may still have affected cytokine levels during both the acute and recovery phases.
5. Short clinical follow-up: Persistent cytokine elevations were documented at 1 month, but longer-term outcomes such as post-acute sequelae (long COVID), pulmonary fibrosis, or quality of life were not assessed.
6. Single follow-up time point: The 1-month evaluation provides only a snapshot of immunological recovery, potentially missing earlier post-discharge dynamics or later convalescent changes.
7. Lack of a healthy control group: Without a non-infected comparator population, it remains uncertain whether cytokine levels at 1 month represented a return to baseline or ongoing immune dysregulation.
8. Restricted respiratory support capacity: The study centers were limited to conventional oxygen delivery (nasal cannula or Hudson-type mask). Non-invasive and invasive mechanical ventilation were not available, restricting direct comparison with cohorts treated in centers offering broader respiratory support.
9. No cytokine–symptom correlation: Although 18% of patients reported persistent symptoms at 1 month, these did not significantly correlate with acute disease severity or cytokine levels. This limits interpretation of the link between immune activation and clinical expression of post-acute sequelae. Larger studies integrating both biological and clinical outcomes will be required to clarify these associations.

## Figures and Tables

**Table 1:** Demographic and clinical characteristics of different COVID-19 patients.

		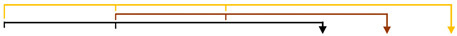					
Characteristics	Total Study Cohort (*n* = 68)	Group 1 (Mild) (*n* = 16)	Group 2 (Moderate) (*n* = 28)	Group 3 (Severe) (*n* = 24)	*p* Value ^1^	*p* Value ^2^	*p* Value ^3^
Age, (years), mean ± SD	69.9 ± 12.4	64.4 ± 9.1	66.2 ± 10.2	69.4 ± 11.3	0.2411	0.1833	0.2134
Male gender, n (%)	40 (58.82%)	10 (62.50%)	16 (57.14%)	14 (58.33%)	0.9768	0.9891	0.9391
BMI ^4^, (kg/m^2^), mean ± SD	29.87 ± 3.9	29.05 ± 3.5	29.93 ± 3.9	29.07 ± 3.4	0.8723	0.7657	0.9133
Hospital stay, (days), mean ± SD	6.7 ± 2.8	4.5 ± 1.9	6.1 ± 2.4	7.9 ± 3.1	<0.05	<0.01	<0.001
Comorbidities:							
Hypertension, n (%)	11 (16.18%)	2 (12.50%)	5 (17.86%)	4 (16.67%)	0.6402	0.9099	0.8949
Dyslipidemia, n (%)	14 (20.59%)	3 (18.75%)	6 (21.43%)	5 (20.83%)	0.8322	0.9582	0.9772
Cardiovascular disease, n (%)	18 (26.47%)	4 (25.00%)	8 (28.57%)	6 (25.00%)	0.7983	0.7722	0.9475
Chronic kidney failure, n (%)	11 (16.18%)	3 (18.75%)	4 (14.29%)	4 (16.67%)	0.6969	0.8125	0.9249
Obesity, n (%)	13 (19.12%)	3 (18.75%)	5 (17.86%)	5 (20.83%)	0.9411	0.7861	0.9628
Diabetes mellitus, n (%)	7 (10.29%)	2 (12.50%)	3 (10.71%)	2 (8.33%)	0.8575	0.7716	0.9096
COPD ^5^, n (%)	15 (22.06%)	4 (25.00%)	6 (21.43%)	5 (20.83%)	0.7857	0.9582	0.9475
Asthma, n (%)	12 (17.65%)	3 (18.75%)	5 (17.86%)	4 (16.67%)	0.9411	0.9099	0.9851
Presenting symptoms:							
Fever, n (%)	58 (85.29%)	12 (75.00%)	25 (89.29%)	21 (87.50%)	0.2127	0.8408	0.4065
Cough, n (%)	24 (35.29%)	5 (31.25%)	12 (42.86%)	7 (29.17%)	0.4469	0.3068	0.5467
Expectoration, n (%)	20 (29.41%)	4 (25.00%)	7 (25.00%)	9 (37.50%)	>0.9999	0.3303	0.5575
Myalgia, n (%)	13 (19.12%)	1 (6.25%)	6 (21.43%)	6 (25.00%)	0.1854	0.7606	0.3093
Diarrhea, n (%)	6 (8.82%)	0 (0.00%)	5 (17.86%)	1 (4.17%)	0.0726	0.1234	0.0806
Physiological variables:							
Respiratory rate, mean ± SD	23.3 ± 2.6	20.1 ± 2.1	22.4 ± 2.5	25.1 ± 3.1	0.0132	0.0090	<0.0001
O_2_ saturation, mean ± SD	92.3 ± 3.9	96.3 ± 2.4	90.1 ± 4.1	82.2 ± 6.4	<0.0001	0.0010	<0.0001

^1^ Group 1 vs. Group 2; ^2^ Group 2 vs. Group 3; ^3^ Overall comparison via one-way ANOVA (for continuous variables, with Tukey’s HSD post hoc test) or Chi-square test (for categorical variables, with Fisher’s exact test and Bonferroni correction applied to pairwise comparisons; α = 0.0167); ^4^ BMI—body mass index; ^5^ COPD—Chronic Obstructive Pulmonary Disease.

**Table 2:** Comparison of circulating inflammatory cytokine levels across subgroups at baseline.

		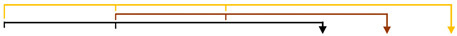					
Characteristics	Total Study Cohort (*n* = 68)	Group 1 (Mild) (*n* = 16)	Group 2 (Moderate) (*n* = 28)	Group 3 (Severe) (*n* = 24)	*p* Value ^1^	*p* Value ^2^	*p* Value ^3^
MIF ^4^, pg/mL, mean ± SD	35,782 ± 14,005	18,896 ± 5202	31,590 ± 5179	51,930 ± 5511	<0.0001	<0.0001	<0.0001
MCP-1 ^5^ (CCL2), pg/mL, mean ± SD	149.7 ± 33.53	150.3 ± 28.69	126.8 ± 15.66	176.1 ± 32.95	0.0010	<0.0001	<0.0001
IP-10 ^6^ (CXCL10), pg/mL, mean ± SD	1213 ± 425.1	648.4 ± 151.7	1180 ± 235.4	1627 ± 209.6	<0.0001	<0.0001	<0.0001
IFN-γ ^7^, pg/mL, mean ± SD	148.2 ± 165.5	4.87 ± 2.58	60.30 ± 20.32	346.3 ± 121.1	<0.0001	<0.0001	<0.0001
IL-4 ^8^, pg/mL, mean ± SD	3.39 ± 0.98	2.64 ± 1.17	3.42 ± 0.68	3.86 ± 0.85	0.0081	0.0414	0.0003
IL-10 ^9^, pg/mL, mean ± SD	6.06 ± 3.97	3.31 ± 1.36	4.12 ± 1.41	10.17 ± 3.86	0.0707	<0.0001	<0.0001
IL-13 ^10^, pg/mL, mean ± SD	5.65 ± 2.91	3.87 ± 1.40	5.08 ± 2.54	7.49 ± 3.13	0.0873	0.0036	0.0001
IL-17 ^11^, pg/mL, mean ± SD	15.25 ± 10.22	5.51 ± 2.91	9.81 ± 2.83	28.07 ± 4.01	<0.0001	<0.0001	<0.0001
TNF-α ^12^, pg/mL, mean ± SD	25.93 ± 20.74	1.21 ± 1.57	18.40 ± 6.26	51.52 ± 6.62	<0.0001	<0.0001	<0.0001

^1^ Group 1 vs. Group 2; ^2^ Group 2 vs. Group 3; ^3^ Overall comparison via one-way ANOVA (for continuous variables, with Tukey’s HSD post hoc test) or Chi-square test (for categorical variables, with Fisher’s exact test and Bonferroni correction applied to pairwise comparisons; α = 0.0167); ^4^ MIF—Macrophage Migration Inhibitory Factor; ^5^ MCP-1 (CCL2)—Monocyte Chemoattractant Protein-1 (Chemokine (C-C motif) Ligand 2); ^6^ IP-10 (CXCL10)—Interferon Gamma-Induced Protein 10 (Chemokine (C-X-C motif) Ligand 10); ^7^ IFN-γ—Interferon-gamma; ^8^ IL-4—Interleukin-4; ^9^ IL-10—Interleukin-10; ^10^ IL-13—Interleukin-13; ^11^ IL-17—Interleukin-17; ^12^ TNF-α—Tumor Necrosis Factor-alpha.

**Table 3:** Comparison of inflammatory cytokine levels across subgroups at 1-month follow-up.

		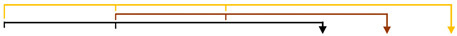					
Characteristics	Total Study Cohort (*n* = 68)	Group 1 (Mild) (*n* = 16)	Group 2 (Moderate) (*n* = 28)	Group 3 (Severe) (*n* = 24)	*p* Value ^1^	*p* Value ^2^	*p* Value ^3^
MIF ^4^, pg/mL, mean ± SD	21,048 ± 8396	11,258 ± 3031	18,084 ± 2782	31,035 ± 2968	<0.0001	<0.0001	<0.0001
MCP-1 ^5^ (CCL2), pg/mL, mean ± SD	45.06 ± 11.01	45.27 ± 9.86	37.37 ± 3.65	53.89 ± 10.96	0.0004	<0.0001	<0.0001
IP-10 ^6^ (CXCL10), pg/mL, mean ± SD	246.6 ± 83.23	126.8 ± 26.71	249.2 ± 46.22	323.5 ± 32.48	<0.0001	<0.0001	<0.0001
IFN-γ ^7^, pg/mL, mean ± SD	22.51 ± 25.12	4.44 ± 2.50	8.71 ± 3.23	50.65 ± 23.16	<0.0001	<0.0001	<0.0001
IL-4 ^8^, pg/mL, mean ± SD	1.46 ± 0.33	1.37 ± 0.55	1.43 ± 0.17	1.56 ± 0.27	0.6373	0.0441	0.1946
IL-10 ^9^, pg/mL, mean ± SD	1.49 ± 0.93	0.98 ± 0.31	0.93 ± 0.31	2.50 ± 0.86	0.6264	<0.0001	<0.0001
IL-13 ^10^, pg/mL, mean ± SD	2.20 ± 0.90	2.13 ± 0.77	2.10 ± 0.96	2.37 ± 0.91	0.8958	0.2957	0.5178
IL-17 ^11^, pg/mL, mean ± SD	7.74 ± 4.20	5.23 ± 2.19	7.70 ± 3.57	9.48 ± 5.08	0.0165	0.1464	0.0059
TNF-α ^12^, pg/mL, mean ± SD	13.62 ± 10.13	1.50 ± 1.00	10.08 ± 3.41	25.83 ± 3.42	<0.0001	<0.0001	<0.0001

^1^ Group 1 vs. Group 2; ^2^ Group 2 vs. Group 3; ^3^ Overall comparison via one-way ANOVA (for continuous variables, with Tukey’s HSD post hoc test) or Chi-square test (for categorical variables, with Fisher’s exact test and Bonferroni correction applied to pairwise comparisons; α = 0.0167); ^4^ MIF—Macrophage Migration Inhibitory Factor; ^5^ MCP-1 (CCL2)—Monocyte Chemoattractant Protein-1 (Chemokine (C-C motif) Ligand 2); ^6^ IP-10 (CXCL10)—Interferon Gamma-Induced Protein 10 (Chemokine (C-X-C motif) Ligand 10); ^7^ IFN-γ—Interferon-gamma; ^8^ IL-4—Interleukin-4; ^9^ IL-10—Interleukin-10; ^10^ IL-13—Interleukin-13; ^11^ IL-17—Interleukin-17; ^12^ TNF-α—Tumor Necrosis Factor-alpha.

## Data Availability

The data presented in this study are available on request from the corresponding author. The data are not publicly available due to privacy reasons.

## Abbreviations

The following abbreviations are used in this manuscript:

COVID-19	Coronavirus Disease 2019
COPD	Chronic Obstructive Pulmonary Disease
SARS-CoV-2	Severe Acute Respiratory Syndrome Coronavirus 2
MIF	Macrophage Migration Inhibitory Factor
MCP-1 (CCL2)	Monocyte Chemoattractant Protein-1 (Chemokine (C-C motif) ligand 2)
IP-10 (CXCL10)	Interferon Gamma-Induced Protein 10 (C-X-C motif chemokine 10)
IFN-γ	Interferon Gamma
IL-4	Interleukin-4
IL-10	Interleukin-10
IL-13	Interleukin-13
IL-17	Interleukin-17
TNF-α	Tumor Necrosis Factor Alpha
ELISA	Enzyme-Linked Immunosorbent Assay
ARDS	Acute Respiratory Distress Syndrome
RT-PCR	Reverse Transcription Polymerase Chain Reaction
ICU	Intensive Care Unit
PASC	Post-Acute Sequelae of COVID-19
BMI	Body Mass Index
SpO_2_	Peripheral Capillary Oxygen Saturation
CD74	Cluster of Differentiation 74 (MIF receptor)
xMAP^®^	Multi-Analyte Profiling (Luminex technology)
SD	Standard Deviation
